# Peripheral autonomic failure is associated with more severe postprandial hypotension compared to central autonomic failure

**DOI:** 10.1007/s10286-025-01131-x

**Published:** 2025-05-13

**Authors:** Pouya E. Mehr, Pedro J. Ortiz, Kaitlyn R. O’Rourke, Tan Ding, Amber J. Hackstadt, Surat Kulapatana, André Diedrich, Daniel O. Claassen, Italo Biaggioni, Amanda C. Peltier, Cyndya A. Shibao

**Affiliations:** 1 Department of Medicine, Division of Clinical Pharmacology, Autonomic Dysfunction Center, Vanderbilt University Medical Center, 2220 Pierce Avenue 506 RRB, Nashville, TN 37027 USA; 2 Department of Neurology, Vanderbilt Center for Cognitive Medicine, Vanderbilt University Medical Center, Nashville, TN USA; 3 Department of Biostatistics, Vanderbilt University Medical Center, Nashville, TN USA; 4 Department of Biomedical Engineering, Vanderbilt University, Nashville, TN USA; 5 Department of Physiology, Faculty of Medicine Siriraj Hospital, Mahidol University, Bangkok, Thailand

**Keywords:** Postprandial hypotension, Autonomic failure, Parkinson disease, Multiple system atrophy

## Abstract

**Purpose:**

Postprandial hypotension (PPH) defined as a decrease in systolic blood pressure of more than 20 mmHg within 2 h post meal is prevalent in patients with autonomic failure and is associated with negative cardiovascular outcomes. Previous studies reported peripheral autonomic failure with less residual sympathetic tone in Parkinson disease (PD). Therefore, we hypothesized that PPH is more severe in PD than in multiple system atrophy (MSA) with central autonomic failure.

**Methods:**

Thirteen patients with PD and 13 patients with MSA were enrolled. Autonomic function testing and neurohormonal measurements were performed to assess autonomic failure and residual sympathetic activity. Subjects were fed a standard breakfast. Systolic and diastolic blood pressure and heart rate were monitored every 5 min from 30 min before to 120 min post meal. Postprandial hemodynamic changes were summarized using area under the curve (AUC). Differences between the groups were assessed with two-sample independent* t* test and linear regression.

**Results:**

Patients with PD (69% male, 72 ± 9 years) had a significantly lower post-meal diastolic blood pressure (*P* = 0.003) and heart rate AUC (*P* = 0.007) than patients with MSA (62% male, 62 ± 8 years). After adjusting for age and supine systolic blood pressure, PD as diagnosis still had significant estimate effect for diastolic blood pressure AUC (*P* = 0.019). No significant difference was found in the mean systolic blood pressure AUC, but at 30 min post meal, systolic blood pressure decrease was significantly lower in PD (*P* = 0.016).

**Conclusion:**

The PD group with peripheral autonomic failure exhibits more severe PPH than the MSA group. This highlights the need for tailored management for PPH in PD.

## Introduction

Postprandial hypotension (PPH) is a condition characterized by a sudden and substantial decline of more than 20 mmHg in systolic blood pressure (SBP) and more than 10 mmHg in diastolic blood pressure (DBP) within 2 h following meal intake [1]. PPH increases the risk of syncope and falls, and in severe cases can cause angina and stroke, especially when presenting chronically over an extended period in elderly patients [2, 3].

Normally after meal consumption, blood flow is redistributed to the splanchnic vascular bed, which causes a decline in blood pressure sensed by baroreceptors of mechanosensitive neurons located in the sinuses of internal carotid artery and the aortic arch [4, 5]. The signals sent by the afferent neurons ultimately trigger disinhibition of the central autonomic commands, increasing sympathetic activation with subsequent vasoconstriction and restoration of the cardiac output. These compensatory mechanisms prevent any significant changes in blood pressure. Therefore, it is not unexpected for PPH to occur in conditions associated with autonomic impairment such as in pure autonomic failure, Parkinson disease (PD), and multiple system atrophy (MSA) among others. 

The accumulation of phosphorylated α-synuclein protein has been repeatedly identified amongst the sources of such autonomic impairment, causing damage of postganglionic axonal neuropathy in PD and in preganglionic neurons in MSA [6–8]. Of note, in MSA postganglionic neuronal pathways are preserved, leading to residual sympathetic activity despite centrally sourced autonomic failure [9–11]. Furthermore, the common clinical presentation of these patients is neurogenic orthostatic hypotension (nOH), which is a substantial decrease in blood pressure upon standing [12], and it is estimated to affect up to approximately 80% of MSA and 58% of patients with PD, respectively [13, 14]. PPH exacerbates nOH putting patients at risk for syncope and complicating its treatment. Since humans spend most of the day in the postprandial state, it is crucial to understand what factors contribute to severity of PPH to then be able to better prevent and treat this condition.

This study’s purpose was to compare the occurrence of PPH between patients with PD and MSA with autonomic failure, seeking to elicit more attention amongst researchers and clinicians in the field to each disorder’s contribution to a comorbidity that can be severely debilitating for these patient populations. While previous studies have looked at prevalence of PPH in various neurological diseases including PD and MSA, our study sought to expand on it by not only specifically focusing on the contribution of peripheral autonomic failure presented through PD compared to central autonomic failure in MSA in PPH severity but also doing so by extensively characterizing the pathophysiology of autonomic failure in these patient groups [15, 16].

We hypothesized that PD is associated with a larger drop in postprandial BP and therefore more severe PPH than MSA due to postganglionic denervation in PD and retention of residual sympathetic activity in MSA, responsible for modulating vascular resistance and myocardial activity [9–11, 17, 18]. Higher sympathetic outflow in patients with MSA, assessed by higher increase in plasma catecholamine levels than in PD, to counteract transient BP and HR changes induced by external stressors was expected to demonstrate retention of sympathetic activity in patients with MSA [19].

## Methods

The study was approved by the Vanderbilt Human Research Protections Program. As a retrospective study, its data were derived from a previous study conducted by the Vanderbilt Autonomic Dysfunction Center that included autonomic function testing and meal challenge as part of baseline evaluations prior to any interventions in the original study, and the data used were only from the patients who met the criteria set for the current study. Participants had been recruited from referrals to the Vanderbilt Autonomic Dysfunction Center between the years 2009 and 2017, and they all had given informed consent for all the procedures prior to the study. For the current study, patients with history of type 1 or type 2 diabetes mellitus, pregnancy or breastfeeding at the time of study inclusion, other secondary causes of autonomic failure, i.e., amyloidosis or autoimmune autonomic ganglionopathy, or any chronic severe cardiovascular disease, renal and liver failure were excluded. All participants had undergone a neurological evaluation by a board-certified movement disorder specialized neurologist to diagnose them with either MSA or PD in alignment with the criteria published by *Neurology* and *JAMA*, respectively [20, 21].

###  Cardiovascular autonomic reflex measurements

Autonomic function tests were performed to determine the integrity of the autonomic reflex arc. The baseline BP and HR were measured while supine and then at 1, 3, 5, 10, 15, 20, 25, and 30 min after standing or until maximum tolerance. In each subject, maximum tolerance was determined to be the onset of presyncopal symptoms during orthostasis that prompted the patient to be moved to the supine position or complete the 30-min mark.

Additionally, blood samples were taken from the subjects while supine and then when they were standing upright to measure catecholamine levels, particularly norepinephrine (NE) and its main intraneural metabolite 3,4-dihydroxyphenylglycol (DHPG), subsequently used to assess sympathetic activity during postural change. Participants were placed supine on a table with their baseline hemodynamic parameters measured. Then, they were asked to stand upright up to 10 min or maximum tolerance. While in supine position, Valsalva maneuver (VM) and sinus arrhythmia (change in HR in response to controlled breathing and orthostatic challenges) were also conducted to assess parasympathetic activity [22, 23]. These tests were previously standardized in our laboratory [24]. Brachial blood pressure and heart rate were measured using an automated brachial sphygmomanometer (Dinamap, GE Medical Systems Information Technologies, Milwaukee, Wis). A Finapress® (BMEYE, Amsterdam, the Netherlands) device was used to record continuous blood pressure using the finger volume clamp method. Data were collected using the WINDAQ (Akron, USA) data acquisition system.

We calculated the changes in HR in response to orthostatic changes in blood pressure as an index of residual sympathetic activity as described by Norcliffe-Kaufmann et al. [25]. Additionally, seven patients (two PD and five MSA) had gone through myocardial scintigraphy with iodine-123* meta*-iodobenzylguanidine (MIBG) to assess sympathetic denervation between the two groups with autonomic failure, following the protocol delineated by Van Vickle and Thompson [26]. Only a subset of subjects underwent plasma catecholamine measurement and MIBG imaging. This was due to random variability in accessibility and individual patient decision, rather than the overall study design.

### Meal challenge study

Patients had been admitted to the Vanderbilt Clinical Research Center and were fed a low-monoamine, caffeine-free diet containing 150 mEq sodium and 70 mEq potassium per day for at least 3 days before evaluation. Medication that affects autonomic nervous system and blood volume including fludrocortisone were withheld for at least five elimination half-lives before admission except for anti-parkinsonian medications. As a result of ethical concerns and the potential risk of increasing rigidity in patients with PD, levodopa/carbidopa were not discontinued throughout the study. None of the subjects with PD were on long-acting dopaminergic medications.

While in the fasting state, patients had been asked to consume a standard mixed meal (450 kcal), consisting of 12% protein, 42% fat, and 46% carbohydrates, constituting the meal challenge [27]. SBP, DBP, and HR were measured for 30 min before to establish baseline hemodynamic measurements and then after the meal challenge in 5-min increments up to 120 min. Measurements had been collected while the patient was seated with their feet up on a bed or recliner.

### Biostatistical analyses

Continuous data were summarized using mean ± SD and categorical data were summarized using percents and counts. The SBP measurements was taken every 5 min for 120 min after the mixed meal and the area under the curve (AUC) created by these measurements was the primary outcome. The secondary outcomes included the AUC for DBP and HR measurements for the same time points as collective measurements of PPH severity along with change in SBP, DBP, and HR 30 min post meal intake. The AUC was calculated using the trapezoidal rule, and the baseline values used in the calculation were found by averaging the measurements before meal consumption. Similar to previous studies that have assessed BP and HR measurements over time across different patient groups, in our study AUC was selected as the primary method of analysis because it provides a cumulative measure of postprandial BP and HR response throughout the 2-h postprandial period [28, 29]. It captures both the magnitude and duration of fluctuations in these parameters in this timeframe of interest, allowing for a comparison of the overall impact of meal consumption on cardiovascular function between patients with PD and MSA with autonomic failure.

Observed differences between PD and MSA groups during the autonomic function testing were also examined to demonstrate and assess the extent of autonomic failure.* Q*–*Q* plots and Shapiro–Wilk test were used to assess the normality assumption. Our sample size of 13 subjects per group had 80% power to detect an effect size of 1.15 for the primary outcome using a two-sample* t *test. Fisher’s exact test was used for comparisons of categorical variables. Additionally, to assess their association with SBP, univariable linear regression analyses between AUC for SBP and the baseline covariates of diagnosis group, age, BMI, sex, supine SBP, SA ratio, ∆HR/∆SBP, VM HR IV, and Valsalva ratio were conducted. Finally, multivariable linear regression analyses were conducted for both the primary outcome of AUC for SBP and other AUC outcomes (DBP and HR) to adjust for potential observed differences and confounding variables between the PD and MSA groups for imbalanced variables between the diagnosis groups. Patients with incomplete data caused by randomly failing to collect were excluded from the univariate and multivariable regression analyses.

To determine the proportion of subjects with PPH in each group, we calculated the nadir in SBP and DBP from baseline across the 2-h post meal for each patient. Each subject’s baseline BP was found by averaging the hemodynamic measurements at each 5-min increment before meal consumption. Patients met PPH criteria if the largest post-meal decline of a subject was at least 20 mmHg (≤ − 20 mmHg) for ∆SBP and at least 10 mmHg for ∆DBP (≤ − 10 mmHg).

## Results

### Study population

Twenty-six patients were recruited, 13 with PD and 13 with probable MSA, and their demographic information and characteristics are presented in Table 1. Patients with PD were significantly older than those with MSA (72 ± 9 vs. 62 ± 8 years old in MSA, *P* = 0.005). In both groups, the majority were male, without any significant difference in BMI. Nine out of 13 subjects with PD who had already been taking levodopa/carbidopa medications upon enrollment were kept on them.

**Table 1:** Demographic data and autonomic function tests results

Demographic data					
Parameters	*N*	Parkinson disease *N* = 13	*N*	Multiple system atrophy *N* = 13	*P*
Age (years)	13	72.5 ± 9.1	13	61.9 ± 8.2	0.005*
Male % (*n*)	13	69.2% (9)	13	61.5% (8)	1.000
Weight (kg)	13	76.8 ± 12.3	13	80.3 ± 17.2	0.551
Height (cm)	13	176.0 ± 12.2	13	171.2 ± 11.2	0.303
BMI (kg/m^2^)	13	24.8 ± 3.3	13	27.6 ± 6.7	0.197
Orthostatic stress test					
Supine SBP (mmHg)	12	172.0 ± 22.2	11	145.0 ± 22.4	0.009*
Standing SBP at 3 min (mmHg)	11	103.0 ± 31.7	9	105.4 ± 20.5	0.842
∆SBP (mmHg)	12	− 75.4 ± 56.8	9	− 44.9 ± 18.4	0.140
Supine DBP (mmHg)	12	87.2 ± 18.1	11	83.5 ± 12.7	0.579
Standing DBP (mmHg) at 3 min	11	61.4 ± 10.7	9	66.4 ± 17.0	0.427
∆DBP (mmHg)	12	− 30.9 ± 29.5	9	− 24.4 ± 24.11	0.582
Supine HR (bpm)	12	63.3 ± 13.3	11	66.2 ± 8.4	0.537
Standing HR at 3 min (bpm)	11	70.3 ± 8.6	9	80.3 ± 11.3	0.036*
∆HR (bpm)	11	6.7 ± 14.9	9	12.7 ± 5.9	0.272
∆HR/∆SBP	10	− 0.223 ± 0.18	9	− 0.365 ± 0.29	0.1780
Sinus arrhythmia and Valsalva maneuver analysis					
Sinus arrhythmia ratio	10	1.05 ± 0.03	11	1.06 ± 0.05	0.517
VM, baseline SBP (mmHg)	10	155.5 ± 27.8	11	153.6 ± 21.6	0.87
VM, baseline HR (bpm)	9	70.2 ± 11.0	11	72.1 ± 6.0	0.655
VM II SBP fall (mmHg)	9	92.1 ± 21.3	11	98.8 ± 23.2	0.513
Depressor response to VM II (∆SBP at VM II) (mmHg)	9	− 72.6 ± 28.4	11	− 54.8 ± 20.0	0.111
VM IV SBP overshoot (mmHg)	9	106.0 ± 35.4	11	133.5 ± 28.4	0.070
BP response to VM IV (∆SBP at VM IV) (mmHg)	9	− 52.6 ± 25.6	11	− 20.2 ± 19.0	0.003*
Valsalva ratio	9	0.98 ± 0.25	10	1.2 ± 0.08	0.027*

Continuous variables summarized with mean ± standard deviation; categorical with percent and counts

*BMI* body mass index, *SBP* systolic blood pressure, *DBP* diastolic blood pressure, *HR* heart rate, *VM* Valsalva maneuver, *∆* change from supine to upright

*Statistically significant (*P* < 0.05)

### Autonomic function testing results

Supine SBP was significantly elevated in PD compared to MSA (172.0 ± 22.2 vs. 145.0 ± 22.4 mmHg in MSA, *P* = 0.009). Sinus arrhythmia ratio was abnormal for both groups (normal > 1.2) [30] and was not significantly different (1.05 ± 0.03 PD vs. 1.06 ± 0.05 MSA, *P* = 0.517). Patients with PD had a significantly lower Valsalva ratio than patients with MSA (0.98 ± 0.25 PD vs. 1.2 ± 0.08 MSA, *P* = 0.027) [23]. There was no difference in the depressor response to phase II VM between groups. Further, there was a blunted pressor response in phase IV VM (− 52.6 ± 25.6 PD vs. − 20.2 ± 19.0 MSA,* P* = 0.003). Patients with MSA had a significantly higher HR within 3 min of standing than patients with PD (80.3 ± 11.3 vs. 70.3 ± 8.6 bpm in PD, *P* = 0.036), but there was no difference in the degree of orthostatic hypotension. There was no significant difference in the ∆HR/∆SBP index between groups (0.223 ± 0.18 bpm/mmHg PD vs. − 0.365 ± 0.29 bpm/mmHg, *P* = 0.178, |normal value|> 0.5), see Table 1.

**Fig. 1:**
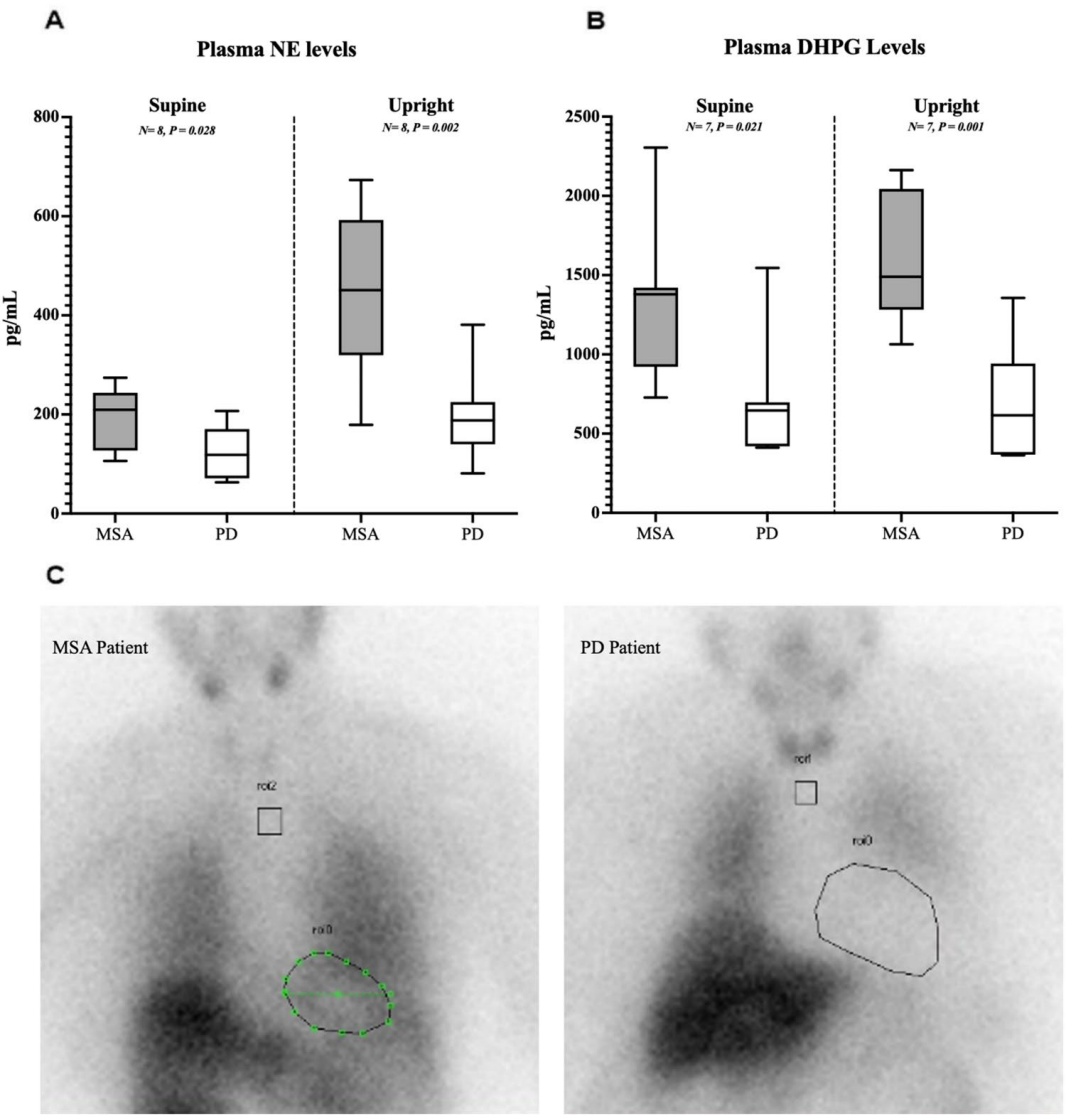
**a** MSA (dark gray) and PD (white) plasma norepinephrine levels supine and upright. **b** MSA (dark gray) and PD (white) plasma DHPG levels supine and upright. **c** MIBG imaging for a patient with MSA and patient with PD patient in the study

There was a significant difference in both NE levels between patients with PD and patients with MSA while supine (122.0 ± 52.3 vs. 193.6 ± 60.9 pmol/L respectively, *P* = 0.028, Fig. 1a) as well as in DHPG (711.0 ± 385.1 vs. 1342.0 ± 5500.1 pmol/L respectively, *P* = 0.021, Fig. 1b). Patients with MSA had a significantly higher plasma NE while upright than patients with PD (448.9 ± 162.3 pmol/L in MSA vs. 229.3 ± 88.2 pmol/L in PD, *P* = 0.002) along with higher DHPG levels (1563.0 ± 351.1 vs. 721.7 ± 305.9 pmol/L in PD, *P* = 0.001). Upon standing, plasma NE and DHPG levels increased significantly more in patients with PD than in patients with MSA (∆NE 255.0 ± 122.0 vs. 69.0 ± 67.3 pmol/L respectively, *P* = 0.003) and (∆DHPG 301.4 ± 186.1 vs. 108.1 ± 86.5 pmol/L in PD, *P* = 0.028).

However, there was not a significant difference in NE-to-DHPG ratio between MSA and PD in supine (0.238 ± 0.085 in PD vs. 0.168 ± 0.06 in MSA, *P* = 0.168), upright (0.309 ± 0.094 in PD vs. 0.312 ± 0.157 in MSA, *P* = 0.976), and the change between the two positions (∆NE/∆DHPG 0.071 ± 0.067 in PD vs. 0.144 ± 0.103 in MSA, *P* = 0.146).

The cardiac MIBG imaging results of a patient with MSA and a patient with PD are depicted in Fig. 1c. The results clearly demonstrate MIBG reuptake, shown with neon green dots, by sympathetic noradrenergic neurons in the heart in MSA, but not in PD. Patients with MSA had a higher and normal average heart-to-mediastinum (H/M) ratio (1.9 ± 0.2) while the H/M ratio in patients with PD fell below the normal value of 1.6 (1.1 ± 0.06 PD) [31].

### Meal challenge results

While the largest postprandial decline in BP (minimum ∆SBP and ∆DBP over the 2-h period) demonstrated all patients with PD (*N* = 13) to have PPH (|Min ∆SBP|> 20 mmHg and |Min ∆DBP|> 10 mmHg), only 69% of patients with MSA (*N* = 9) met this definition for PPH [1]. The median largest postprandial SBP decline (minimum ∆SBP) in patients with PD was − 41.7 mmHg (IQR − 56.7, − 33.3 mmHg) while that in patients with MSA − 28.6 mmHg (IQR − 40.7, − 17.9 mmHg). Additionally, the median of the largest postprandial DBP decrease for patients with PD (minimum ∆DBP) was − 22.7 mmHg (IQR − 27.7, − 15.9 mmHg) whereas for MSA the median was − 19.6 mmHg (IQR − 22.6, − 14.2 mmHg).

**Fig. 2:**
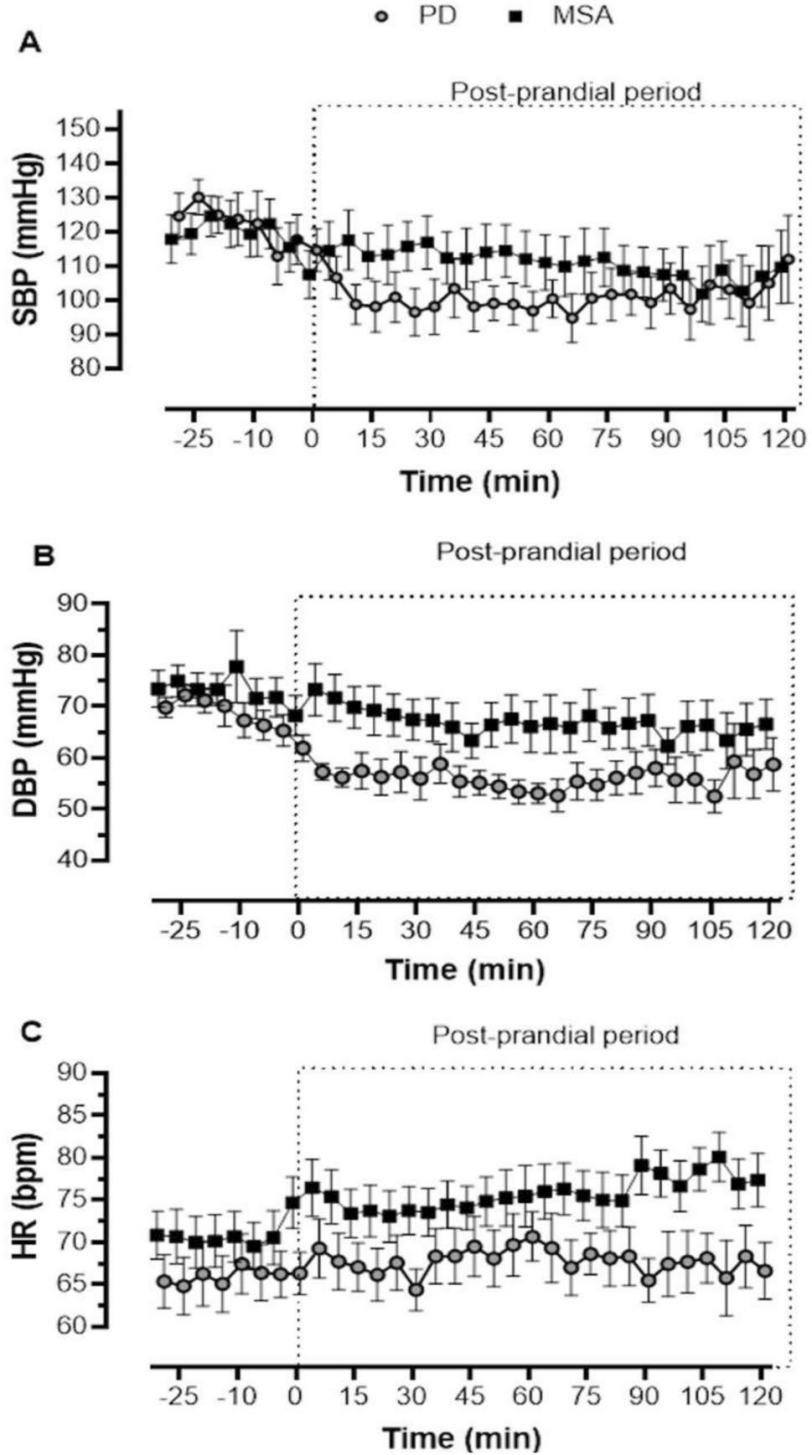
**a** Average SBP, **b** average DBP, and **c** average HR values for PD (open gray circles) and MSA (black squares) during the meal challenge at 5-min increments from 30 min before (signified with negative values) to 120 min after meal intake

The normality assumption for the primary and secondary endpoints assessing the severity of PPH between the two groups was not violated, as indicated by * Q*–*Q* plots and Shapiro–Wilk tests, namely postprandial SBP AUC (*P* = 0.446), postprandial DBP AUC (*P* = 0.818), and postprandial HR AUC (*P* = 0.345). Although the difference in the AUC values for SBP between groups was not statistically significant (*P* = 0.053), the AUC of SBP was higher for the MSA group (Table 2), and the time-dependent changes in SBP throughout the meal challenge are shown in Fig. 2a. There was, however, a significant compounded decline in DBP in patients with PD compared with MSA (1110.3 ± 305.6 vs. 1567.5 ± 398.17 mmHg min in MSA,* P* = 0.003, Table 2) with Fig. 2b showing the time-dependent changes in DBP during the meal challenge. Additionally, HR significantly increased post meal in MSA, but not in PD (AUC of HR 1361.6 ± 373.1 vs. 1762.5 ± 315.5 mmHg min in MSA, *P* = 0.007, Table 2). See time-dependent HR changes in Fig. 2c.

**Table 2:** Unadjusted postprandial hemodynamics analysis

Parameter	*N*	Parkinson disease (mean ± SD)	*N*	Multiple system atrophy (mean ± SD)	*P*
Postprandial SBP AUC (mmHg min)	13	2081.4 ± 605.6	13	2599.0 ± 685.7	0.053
Postprandial DBP AUC (mmHg min)	13	1110.3 ± 305.6	13	1576.5 ± 398.1	0.003*
Postprandial HR AUC (mmHg min)	13	1361.6 ± 373.1	13	1762.5 ± 315.5	0.007*
∆SBP at 30 min post meal (mmHg)	12	− 24.9 ± 24.2	13	− 3.3 ± 15.1	0.016*
∆DBP at 30 min post meal (mmHg)	13	− 9.67 ± 9.9	13	6.3 ± 7.9	0.46
∆HR at 30 min post meal (bpm)	13	− 2.17 ± 13.4	13	3.4 ± 4.7	0.260

*AUC* area under the curve, *SBP* systolic blood pressure, *HR* heart rate, *DBP* diastolic blood pressure, ∆ postprandial change in hemodynamic variables compared to baseline

*Statistically significant (*P* < 0.05)

Moreover, as outlined in Table 2, there was a significantly larger decrease in SBP for patients with PD at 30 min post meal than MSA compared to the baseline values (− 24.9 ± 24.2 vs. − 3.3 ± 15.1 mmHg in MSA, *P* = 0.013), but the decline in DBP 30 min after the meal was not significantly different (− 13.0 ± 12.9 PD vs. 6.3 ± 7.9 mmHg MSA, *P* = 0.460). The median change in SBP at 30 min for PD was − 17.8 (interquartile range (IQR) − 35.8, − 11.3) and median change in DBP at 30 min was − 8.6 (IQR − 15.1, − 6.9) while for the patients with MSA, the a median change in SBP at 30 min was − 3.1 (IQR − 14.6, 8.4) and median change in DBP at 30 min of − 8.4 (IQR − 9.7, − 3.3). There was not a significant difference between the two in HR change 30 min post meal consumption (*P* = 0.205).

### Regression analysis

The univariable linear regression tests between AUC of post-meal SBP and age, BMI, sex, supine SBP, sinus arrhythmia ratio, the index ∆HR/∆BP and HR during phase IV of VM did not show any significant association, Table 3; however, the diagnosis (MSA or PD) had a borderline significant association with AUC of post-meal SBP (*P* = 0.05). Moreover, the results of the multivariable linear regression analysis with independent variables of diagnosis group (PD vs. MSA), age, mean supine SBP, and dependent variable AUC for SBP are shown in Table 3. Of note, five patients (three MSA and two PD) were excluded because of incomplete data. The effect size for PD versus MSA for the primary SBP outcome is relatively large in magnitude (− 664.1, CI − 1544.2, 216.0) with a smaller *p* value (0.130) but larger than the significance level of 0.05. We see similar results for the secondary outcome of the AUC of HR (− 243.3, CI − 551.1, 64.6, *P* = 0.114). However, there was a significant effect estimate for diagnosis groups for the adjusted analysis for AUC of DBP (− 552.4, CI 1001.2, − 103.6, *P* = 0.019).

**Table 3:** Linear regression analyses for AUC of post-meal SBP

Univariable analysis				
Independent variable	Slope	y-intercept	* R*^2^	*P*
Diagnosis (reference MSA)	− 517.6	2599.0	0.15	0.05
Age (years)	− 12.60	3187.1	0.034	0.37
BMI	45.09	1160.09	0.12	0.08
Sex	263.5	2167.9	0.03	0.36
Supine SBP (mmHg)	− 2.29	2671.8	0.008	0.70
Sinus arrhythmia ratio	− 855.4	3346.2	0.002	0.83
∆HR/∆SBP	− 327.4	2261.7	0.037	0.46
VM HR IV (bpm)	0.032	− 13.2	0.001	0.889

Adjusted analysis						
Vs	Diagnosis (MSA or PD)		Age (year)		Supine SBP (mmHg)	
	Estimate (CI)	*P*	Estimate (CI)	*P*	Estimate (CI)	*P*
Post-meal SBP AUC (mmHg min)	− 664.1 (− 1544.2, 216.0)	0.130	− 2.8 (− 42.1, 36.4)	0.881	4.8 (− 9.0, 18.8)	0.470
Post-meal DBP AUC (mmHg min)	− 552.4 (− 1002.2, − 103.6)	0.019*	− 0.9 (− 22.8, 21.0)	0.936	4.3 (− 8.1, 16.7)	0.472
Post-meal HR AUC (beats)	− 243.3 (− 551.1, 64.4)	0.114	6.9 (− 9.1, 22.9)	0.378	32.5 (16.7, 48.4)	< 0.001*

*R*^2^ = 0.20, 0.39, 0.610 for SBP, DBP, HR models, respectively

*SBP* systolic blood pressure, *DBP* diastolic blood pressure, *HR* heart rate, *VM* Valsalva maneuver, AUC area under curve, *PD* Parkinson disease, *MSA* multiple system atrophy, *CI* confidence interval

*Statistically significant (*P* < 0.05)

## Discussion

The main finding of this study is that patients with PD and peripheral autonomic failure have a more substantial decrease in blood pressure after a mixed meal challenge compared with patients with MSA and central autonomic failure. Thus, we suggest continuous screening for PPH occurrence, particularly in patients with PD.

All patients with PD had PPH, whereas only 69% of patients with MSA met PPH criteria. Patients with PD experienced a more severe decline in postprandial AUC DBP than patients with MSA. It is noteworthy that postprandial AUC SBP was different between the two groups; however, when determining the decrease in SBP at 30 min post meal, patients with PD indeed experienced a larger decline than the MSA group.

It is well known that changes in DBP mostly reflect changes in total peripheral resistance, which is highly modulated by sympathetic vasoconstriction activity. Thus, it could be inferred that the decreased residual sympathetic activity in PD plays a role in the observed differences in DBP but not in SBP, further supporting our hypothesis [17, 32, 33]. Patients with MSA had a significant overall increase in HR after meal intake as well, which was more noticeable later in the postprandial period.

Since the two groups differed significantly in terms of age and supine SBP, we performed an adjusted analysis for the primary endpoints AUC SBP and AUC DBP using a multivariable linear regression model. It is noteworthy that the postprandial fall in DBP remained significantly associated with PD, which suggests that PD diagnosis may be a risk factor for PPH.

Autonomic function testing in both groups showed significant autonomic neuropathy affecting both sympathetic and parasympathetic function. Sympathetic vasoconstrictor activity was blunted, as noted by the absence of an increase in SBP in phase II and phase IV of the VM [34]. While the Valsalva ratio was below normal for both, MSA’s significantly higher ratio indicated a lower degree of parasympathetic impairment compared to PD.

Upon changing to the upright position, patients with MSA experienced a significantly higher increase in both NE and DHPG levels compared to patients with PD. The NE/DHPG ratio, as a function of norepinephrine transporter (NET) reuptake activity, was not different between groups, which supports the presence of residual sympathetic activity in MSA due to sympathetic outflow rather than differences in NET reuptake activity [35, 36].

Furthermore, in a subset of patients, the results of the myocardial scintigraphy with MIBG demonstrated sympathetic denervation in the heart in patients with PD as indicated by lower-than-normal heart-mediastinum (H/M) ratio compared to MSA [31, 37]. Higher H/M in patients with MSA demonstrated that postganglionic sympathetic neurons and their axons innervating the heart were preserved in this group as opposed to PD [38, 39]. The residual sympathetic activity tonically regulates blood pressure in MSA because previous studies have demonstrated that blocking this activity with pharmacological agents such as clonidine and trimethaphan results in a decrease in blood pressure in this group [40].

In subjects with healthy autonomic functioning, hemodynamic measurements reported after meal consumption showed a 62% increase in cardiac output, which is attributable to a 17% increase in HR and 41% increase in stroke volume, counteracting the decline in BP caused by splanchnic blood pooling [41]. Furthermore, since the source of autonomic impairment in MSA is preganglionic and residual sympathetic activity is still intact, it is possible that the increase in HR during the postprandial period mitigates the severity of postprandial, or generally speaking post-strain, decline in this patient group.

As expected, patients with PD were older and had significantly higher supine hypertension compared to patients with MSA. These factors are not indicators of disease severity because both groups had complete autonomic impairment as measured by autonomic testing. The adjusted analysis showed that PD, independent of age and supine hypertension, had a substantial fall in postprandial DBP compared to MSA, indicating that these factors did not contribute to the observed differences.

Finally, 9 out of 13 patients with PD continued with levodopa/carbidopa treatment in the study. Of note, Mehagnoul-Schipper et al. [2] demonstrated that administration of 125 mg of levodopa/benserazide (DOPA decarboxylase inhibitor) twice a day did not contribute to the severity of BP decline in orthostatic and postprandial hypotension; however, other studies have reported that levodopa and carbidopa have vasodilatory and negative inotropic effects, specifically worsening OH in PD with autonomic failure [42, 43]. Therefore, we cannot completely rule out the contribution of these medications to PPH in PD. Our study was not powered for an analysis adjusting for the effect of continuing levodopa/carbidopa, which is a limitation in this study. Thus, caution should be used when generalizing the adjusted analysis to other populations because of the study’s small sample size as overfitting could occur. Nevertheless, discontinuing antiparkinsonian medications is not an option in most patients with PD; therefore, we still recommend screening and treating patients with PD for PPH.

Nonetheless, it should be noted that PPH is a widely overlooked and underdiagnosed condition in PD and MSA patient populations; therefore, we aim for our results, which inform on the pathophysiology of PPH, to bring more attention to this condition amongst researchers and clinicians. In conclusion, our study is the first to report that even within the group of patients with severe autonomic failure, there are differences in PPH occurrence with patients with PD having more severe PPH compared with those with MSA.

## Limitations

This study was small in scale, so caution should be used when generalizing the results to different patient populations as previously discussed, and supports the need for more research on factors that contribute to PPH severity in patients with different types of autonomic failure. This study did not use the 2022 MDS MSA criteria [44] because the enrollment ended in 2017. Moreover, we did not assess how the progression of autonomic failure over time in both patient groups can contribute to the severity of PPH; therefore, a future longitudinal study should address such factors. Finally, other factors such as differences in the effect of vasodilatory hormones in the splanchnic circulation that could contribute to the severity of PPH were not assessed.

## Data Availability

The data that support the findings of this study are available on request.

## Abbreviations

AUC: Area under the curve
BP: Blood pressure
DBP: Diastolic blood pressure
DHPG: 3,4-Dihydroxyphenylglycol
HR: Heart rate
MIBG: *meta*-Iodobenzylguanidine
MSA: Multiple system atrophy
NE: Norepinephrine
NET: Norepinephrine transporter
OH: Orthostatic hypotension
PD: Parkinson disease
PPH: Postprandial hypotension
SBP: Systolic blood pressure
VM: Valsalva maneuver

## References

[1] Jansen RW, Lipsitz LA (1995) Postprandial hypotension: epidemiology, pathophysiology, and clinical management. Ann Intern Med 122:286–295. 10.7326/0003-4819-122-4-199502150-00009 7825766 10.7326/0003-4819-122-4-199502150-00009

[2] Mehagnoul-Schipper DJ, Boerman RH, Hoefnagels WH, Jansen RW (2001) Effect of levodopa on orthostatic and postprandial hypotension in elderly Parkinsonian patients. J Gerontol A Biol Sci Med Sci 56(12):M749–M755. 10.1093/gerona/56.12.M749 11723148 10.1093/gerona/56.12.m749

[3] Aronow WS, Ahn C (1997) Association of postprandial hypotension with incidence of falls, syncope, coronary events, stroke, and total mortality at 29-month follow-up in 499 older nursing home residents. J Am Geriatr Soc 45(9):1051–1053. 10.1111/j.1532-5415.1997.tb05965.x 9288010 10.1111/j.1532-5415.1997.tb05965.x

[4] Pirahanchi Y, Bordoni B (2023) Anatomy, head and neck: carotid baroreceptors. StatPearls, Treasure Island (FL) 30725908

[5] Koffert J, Honka H, Teuho J, Kauhanen S, Hurme S, Parkkola R, Oikonen V, Mari A, Lindqvist A, Wierup N et al (2017) Effects of meal and incretins in the regulation of splanchnic blood flow. Endocr Connect 6:179–187. 10.1530/EC-17-0015 28258126 10.1530/EC-17-0015 PMC5428912

[6] Trahair LG, Kimber TE, Flabouris K, Horowitz M, Jones KL (2016) Gastric emptying, postprandial blood pressure, glycaemia and splanchnic flow in Parkinson’s disease. World J Gastroenterol 22:4860–4867. 10.3748/wjg.v22.i20.4860 27239112 10.3748/wjg.v22.i20.4860 PMC4873878

[7] Fukushima T, Asahina M, Fujinuma Y, Yamanaka Y, Katagiri A, Mori M, Kuwabara S (2013) Role of intestinal peptides and the autonomic nervous system in postprandial hypotension in patients with multiple system atrophy. J Neurol 260:475–483. 10.1007/s00415-012-6660-x 22983428 10.1007/s00415-012-6660-x

[8] Iodice V, Low DA, Vichayanrat E, Mathias CJ (2011) Cardiovascular autonomic dysfunction in MSA and Parkinson’s disease: similarities and differences. J Neurol Sci 310:133–138. 10.1016/j.jns.2011.07.014 21849175 10.1016/j.jns.2011.07.014

[9] Jordan J, Shibao C, Italo B (2015) Multiple system atrophy: using clinical pharmacology to reveal pathophysiology. Clin Auton Res 25(1):53–59. 10.1007/s10286-015-0271-4 25757803 10.1007/s10286-015-0271-4 PMC4431657

[10] Micieli G, Tassorelli C, Martignoni E, Marcheselli S, Rossi F, Nappi G (1995) Further characterization of autonomic involvement in multiple system atrophy: a pupillometric study. Funct Neurol 10:273–80 8837991

[11] Asahina M, Vichayanrat E, Low DA, Idoice V, Mathias CJ (2013) Autonomic dysfunction in parkinsonian disorders: assessment and pathophysiology. J Neurol Neurosurg Psychiatry 84(6):674–680. 10.1136/jnnp-2012-303135 22942216 10.1136/jnnp-2012-303135

[12] Gibbons CH, Schmidt P, Biaggioni I, Frazier-Mills C, Freeman R, Isaacson S, Karabin B, Kuritzky L, Lew M, Low P et al (2017) The recommendations of a consensus panel for the screening, diagnosis, and treatment of neurogenic orthostatic hypotension and associated supine hypertension. J Neurol 264:1567–1582. 10.1007/s00415-016-8375-x 28050656 10.1007/s00415-016-8375-x PMC5533816

[13] Sun Z, Jia D, Shi Y, Hou X, Yang X, Guo J, Li N, Wang J, Sun Q, Zhang H et al (2016) Prediction of orthostatic hypotension in multiple system atrophy and Parkinson disease. Sci Rep. 10.1038/srep21649 26867507 10.1038/srep21649 PMC4751507

[14] Velseboer DC, de Haan RJ, Wieling W, Goldstein DS, de Bie RMA (2011) Prevalence of orthostatic hypotension in Parkinson’s disease: a systematic review and meta-analysis. Parkinsonism Relat Disord 17(10):724–729. 10.1016/j.parkreldis.2011.04.016 21571570 10.1016/j.parkreldis.2011.04.016 PMC5199613

[15] Pavelić A, Krbot Skorić M, Crnošija L, Habek M (2017) Postprandial hypotension in neurological disorders: systematic review and meta-analysis. Clin Auton Res 27(4):263–271. 10.1007/s10286-017-0440-8 28647892 10.1007/s10286-017-0440-8

[16] Trahair LG, Horowitz M, Jones KL (2014) Postprandial hypotension: a systematic review. J Am Med Dir Assoc 15(6):394–409. 10.1016/j.jamda.2014.01.011 24630686 10.1016/j.jamda.2014.01.011

[17] Joyner MJ, Charkoudian N, Wallin BG (2010) Sympathetic nervous system and blood pressure in humans: individualized patterns of regulation and their implications. Hypertension. 10.1161/HYPERTENSIONAHA.109.140186 20497993 10.1161/HYPERTENSIONAHA.109.140186 PMC2891078

[18] Charkoudian N, Rabbitts JA (2009) Sympathetic neural mechanisms in human cardiovascular health and disease. Mayo Clin Proc 84(9):822–830. 10.4065/84.9.822 19720780 10.4065/84.9.822 PMC2735432

[19] Goldstein DS, McCarty R, Polinsky RJ, Kopin IJ (1983) Relationship between plasma norepinephrine and sympathetic neural activity. Hypertension. 10.1161/01.hyp.5.4.552 6345364 10.1161/01.hyp.5.4.552

[20] Gilman S, Wenning GK, Kaufmann H et al (2008) Second consensus statement on the diagnosis of multiple system atrophy. Neurology 71(9):670–676. 10.1212/01.wnl.0000324625.00404.15 18725592 10.1212/01.wnl.0000324625.00404.15 PMC2676993

[21] Gelb DJ, Oliver E, Gilman S (1999) Diagnostic criteria for Parkinson disease. Arch Neurol. 10.1001/archneur.56.1.33 9923759 10.1001/archneur.56.1.33

[22] Sun L, Chen W, Chen Z et al (2020) Dual effect of the Valsalva maneuver on autonomic nervous system activity, intraocular pressure, Schlemm’s canal, and iridocorneal angle morphology. BMC Ophthalmol 20(1):5–5. 10.1186/s12886-019-1275-y 31900115 10.1186/s12886-019-1275-y PMC6942388

[23] Novak P (2011) Quantitative autonomic testing. J Visual Exp. 10.3791/2502 10.3791/2502 PMC3196175 21788940

[24] Singer W, Low P (2023) Autonomic function testing. In: Biaggioni I, Browning K, Fink G, Jordan J, Low P, Paton J (eds) Primer on the autonomic nervous system. Academic, San Diego, p 379

[25] Norcliffe-Kaufmann L, Kaufmann H, Palma JA, Shibao CA, Biaggioni I, Peltier AC, Singer W, Low PA, Goldstein DS, Gibbons CH et al (2018) Orthostatic heart rate changes in patients with autonomic failure caused by neurodegenerative synucleinopathies. Ann Neurol 83:522–531. 10.1002/ana.25170 29405350 10.1002/ana.25170 PMC5867255

[26] Van Vickle SS, Thompson RC (2015) 123I-MIBG imaging: patient preparation and technologist’s role. J NuclMed Technol. 10.2967/jnmt.115.158394 10.2967/jnmt.115.158394 25956690

[27] Mayo Clinic Staff (2023) Carbohydrates: how carbs fit into a healthy diet. Mayo Clinic Health Information Library. Tribune Content Agency LLC

[28] Lodhi HA, Peri-Okonny PA, Schesing K et al (2019) Usefulness of blood pressure variability indices derived from 24-hour ambulatory blood pressure monitoring in detecting autonomic failure. J Am Heart Assoc 8(7):e010161–e010161. 10.1161/JAHA.118.010161 30905258 10.1161/JAHA.118.010161 PMC6509738

[29] Okamoto LE, Shibao C, Gamboa A et al (2012) Synergistic effect of norepinephrine transporter blockade and α-2 antagonism on blood pressure in autonomic failure. Hypertension 59(3):650–656. 10.1161/HYPERTENSIONAHA.111.184812 22311903 10.1161/HYPERTENSIONAHA.111.184812 PMC3312003

[30] Naschitz JE, Slobodin G, Elias N, Rosner I (2006) The patient with supine hypertension and orthostatic hypotension: a clinical dilemma. Postgrad Med J 82(966):246–253. 10.1136/pgmj.2005.037457 16597811 10.1136/pgmj.2005.037457 PMC2579630

[31] Muxí Á, Paredes P, Navales I et al (2011) Diagnostic cutoff points for 123I-MIBG myocardial scintigraphy in a Caucasian population with Parkinson’s disease. Eur J Nucl Med Mol Imaging 38(6):1139–1146. 10.1007/s00259-011-1754-8 21373765 10.1007/s00259-011-1754-8

[32] Shahoud J, Sanvictores T, Aeddula N (2023) Physiology, aretrial pressure regulation. StatPearls, Treasure Island (FL) 30860744

[33] Ranjan A, Gulati A (2023) Controls of central and peripheral blood pressure and hemorrhagic/hypovolemicshock. J Clin Med. 10.3390/jcm12031108 36769755 10.3390/jcm12031108 PMC9917827

[34] Srivastav S, Jamil R (2023) Valsalva maneuver. StatPearls, Treasure Island (FL) 30725933

[35] Goldstein D (2024) Autonomic medicine for students. Ebook. https://research.ninds.nih.gov/sites/default/files/documents/Autonomic%20Medicine...for%20Students%20dg%202-15-2024-R.pdf

[36] Goldstein D, Cheshire W (2018) Roles of catechol neurochemistry in autonomic function testing. Clin Auton Res 28(3):273–288. 10.1007/s10286-018-0528-9 29705971 10.1007/s10286-018-0528-9 PMC8895275

[37] Goldstein DS, Holmes C, Li ST, Bruce S, Metman LV, Cannon RO (2000) Cardiac sympathetic denervation in Parkinson disease. Ann Intern Med 133:338–347. 10.7326/0003-4819-133-5-200009050-00009 10979878 10.7326/0003-4819-133-5-200009050-00009

[38] Orimo S, Ozawa E, Oka T, Nakade S, Tsuchiya K, Yoshimoto M, Wakabayashi K, Takahashi H (2001) Different histopathology accounting for a decrease in myocardial MIBG uptake in PD and MSA. Neurology 57(6):1140–1141. 10.1212/wnl.57.6.1140 11571358 10.1212/wnl.57.6.1140

[39] Braune S, Reinhardt M, Schnitzer R, Riedel A, Lücking CH (1999) Cardiac uptake of [123I]MIBG separates parkinson’s disease from multiple system atrophy. Neurology 53(5):1020–1025. 10.1212/wnl.53.5 10496261 10.1212/wnl.53.5.1020

[40] Jordan J, Shibao C, Biaggioni I (2015) Multiple system atrophy: using clinical pharmacology to reveal pathophysiology. Clin Auton Res. 25:53–59. 10.1007/s10286-015-0271-4 25757803 10.1007/s10286-015-0271-4 PMC4431657

[41] Waaler BA, Eriksen M, Toska K (1991) The effect of meal size on postprandial increase in cardiac output. Acta Physiol Scand 142:33–39. 10.1111/j.17481716.1991.tb09125.x 1877363 10.1111/j.1748-1716.1991.tb09125.x

[42] Noack C, Schroeder C, Heusser K, Lipp A (2014) Cardiovascular effects of levodopa in Parkinson’s disease. Parkinsonism Relat Disord 20(8):815–818. 10.1016/j.parkreldis.2014.04.007 24819390 10.1016/j.parkreldis.2014.04.007

[43] Cani I, Guaraldi P, Giannini G et al (2024) Levodopa-induced orthostatic hypotension in parkinsonism: a red flag of autonomic failure. Eur J Neurol. 10.1111/ene.16061 37724992 10.1111/ene.16061 PMC11235727

[44] Wenning GK, Stankovic I, Vignatelli L et al (2022) The Movement Disorder Society criteria for the diagnosis of multiple system atrophy. Mov Disord 37(6):1131–1148. 10.1002/mds.29005 35445419 10.1002/mds.29005 PMC9321158

